# Role of methylation in vernalization and photoperiod pathway: a potential flowering regulator?

**DOI:** 10.1093/hr/uhad174

**Published:** 2023-08-29

**Authors:** Meimei Shi, Chunlei Wang, Peng Wang, Fahong Yun, Zhiya Liu, Fujin Ye, Lijuan Wei, Weibiao Liao

**Affiliations:** College of Horticulture, Gansu Agricultural University, Lanzhou 730070, China; College of Horticulture, Gansu Agricultural University, Lanzhou 730070, China; Vegetable and Flower Research Institute, Chinese Academy of Agricultural Sciences, Beijing 100081, China; College of Horticulture, Gansu Agricultural University, Lanzhou 730070, China; College of Horticulture, Gansu Agricultural University, Lanzhou 730070, China; College of Horticulture, Gansu Agricultural University, Lanzhou 730070, China; College of Horticulture, Gansu Agricultural University, Lanzhou 730070, China; College of Horticulture, Gansu Agricultural University, Lanzhou 730070, China

## Abstract

Recognized as a pivotal developmental transition, flowering marks the continuation of a plant’s life cycle. Vernalization and photoperiod are two major flowering pathways orchestrating numerous florigenic signals. Methylation, including histone, DNA and RNA methylation, is one of the recent foci in plant development. Considerable studies reveal that methylation seems to show an increasing potential regulatory role in plant flowering via altering relevant gene expression without altering the genetic basis. However, little has been reviewed about whether and how methylation acts on vernalization- and photoperiod-induced flowering before and after **FLOWERING LOCUS C* (*FLC*)* reactivation, what role RNA methylation plays in vernalization- and photoperiod-induced flowering, how methylation participates simultaneously in both vernalization- and photoperiod-induced flowering, the heritability of methylation memory under the vernalization/photoperiod pathway, and whether and how methylation replaces vernalization/photoinduction to regulate flowering. Our review provides insight about the crosstalk among the genetic control of the flowering gene network, methylation (methyltransferases/demethylases) and external signals (cold, light, sRNA and phytohormones) in vernalization and photoperiod pathways. The existing evidence that RNA methylation may play a potential regulatory role in vernalization- and photoperiod-induced flowering has been gathered and represented for the first time. This review speculates about and discusses the possibility of substituting methylation for vernalization and photoinduction to promote flowering. Current evidence is utilized to discuss the possibility of future methylation reagents becoming flowering regulators at the molecular level.

## Introduction

Flowering is an integrated event converging multiple internal and external signals. The internal signals mainly involve a number of flowering regulators and endogenous phytohormones that influence floral transition. These flowering regulators are mainly divided into two categories: floral integrators and characteristic meristematic genes. Floral integrators mainly include four types, namely *FLOWERING LOCUS T* (*FT*), *CONSTANS*-*LIKE* (*COL*), *SUPPRESSOR OF OVEREXPRESSION OF CO1* (*SOC1*), and *FLOWERING LOCUS C* (*FLC*), and characteristic meristem genes mainly include five types, namely *LEAFY* (*LFY*), *AGAMOUS*-*LIKE* (*AGL*), *APETALA 1* (*AP1*), *CAULIFLOWER* (*CAL*), and *SEPALLATA* (*SEP*). These integrator genes in flowering pathways trigger floral transition and activate a range of floral meristem identity (FMI) genes. The proteins encoded by FMI genes boost floral development by promoting flower development genes as well as by repressing AGL24, a promoter of inflorescence fate [1]. The main pathways orchestrating blossoming mediated by the internal and external signals can be divided into several types: the floral inhibition pathway [2], the autonomous pathway [3], the photoperiod pathway [4], the vernalization pathway [5], the gibberellin (GA) pathway [6], the stress pathway [7] and the aging pathway [8].

Methylation is a crucial concept in epigenetic mechanisms (acetylation, methylation, RNA interference). As a key factor in the evolution and adaptation of plants, methylation is involved in almost every stage of plant development. As the covalent methylation modification of the fifth cytidine site, DNA methylation is the most well-known and studied epigenetic mechanism in plants. CHH, CG, and CHG (where H represents a base, A/T/C) are three DNA methylation contexts. DNA methylation regulates temporal and spatial gene expression and condition-dependent phenotypic traits, including changing flower symmetry [9] or phenotypic plasticity [10] in different biological processes [11]. Histone methylation, as a vital and reversible post-translational modification (PTM), is one of the most important modifications on histone lysine (K) and arginine (R) residues and regulates many crucial biological processes [12]. RNA methylation is an important modification in plant development and the abiotic stress response. Methylation on RNA bases, such as N^6^-methyladenosine (m^6^A) and 5-methylcytidine (m^5^C), is the most ubiquitous mRNA modification in eukaryotes [13]. The role of methylation in vernalization and photoperiod pathways has been gradually revealed in recent years.

Plant vernalization is an adaptive characteristic acquired in response to chilling temperature, in order to bypass cold seasons and promote selective flowering in spring when temperatures are more favorable. Some advances have been reviewed on the molecular mechanism of chromatin modification indicating that vernalization, as an epigenetic switch for silencing *FLC* and five *FLC* relatives, *MADS AFFECTING FLOWERING 1/2/3/4/5* (*MAF1/2/3/4/5*), promotes *Arabidopsis thaliana* flowering [14, 15]. Alexandre and Hennig discussed progress on the *FLC*/*FLC* branch-independent vernalization pathway in *Arabidopsis* and grasses, and reviewed epigenetic mechanisms of the flowering promoter AGL24 and inhibitor *MAF* involved in vernalization of *Arabidopsis* [16]. In many species, duration of the light–dark cycle (photoperiod) strongly affects flowering. The effects of photoperiod on plants with different light requirements are very different. Notably, the genetic and epigenetic mechanisms (deacetylation and chromatin modification) of seasonal timing of *Arabidopsis* flowering have been summarized [17]. One review mentioned that chromatin modifiers can regulate photoperiodic flowering, but the specific chromatin modifiers have not been included [18]. The interactive roles of chromatin modification and the circadian clock have been elaborated in the review by Chen and Mas [19]. DNA methylation in vernalization has been described [20], but its roles in photoperiod have not been overviewed. Roles of RNA methylation in the vernalization and photoperiod pathways have not been reviewed yet.

In this review we summarize the molecular evidence that histone, DNA, and RNA methylation are involved in flowering regulation in the vernalization and photoperiod pathways. We expound the intricate molecular regulation network among flowering genes, methylation (methyltransferases/demethylases), sRNAs and phytohormone and environmental signals (cold and light). Then, we show the molecular evidence that methylation is involved simultaneously in both vernalization- and photoperiod-induced flowering. The existing evidence for the flowering regulation of RNA methylation in the vernalization and photoperiod flowering pathway is collected for the first time. The heritability of methylation state combined with vernalization and photoperiod memory is discussed. Finally, this review speculates that methylation as a flowering accelerator/inhibitor may substitute for vernalization or photoinduction of flowering, and considers the feasibility of realizing this substitutability in the future.

## Methylation is involved in vernalization-induced flowering

A large proportion of plants need continuous low temperature (vernalization) to fulfill their crucial development transition so that plants enter reproductive growth from vegetative growth. Especially for the absolute vernalization plants, low temperature is a must. Recent studies show that methylation may play an important role in the vernalization-induced flowering pathway. Histone methylation modification, DNA methylation and RNA methylation may alter when flowering plants are undergoing vernalization.

### Histone methylation of lysine and arginine pathways in the vernalization pathway and the potential substitution of histone lysine methylation for vernalization

**Figure 1:**
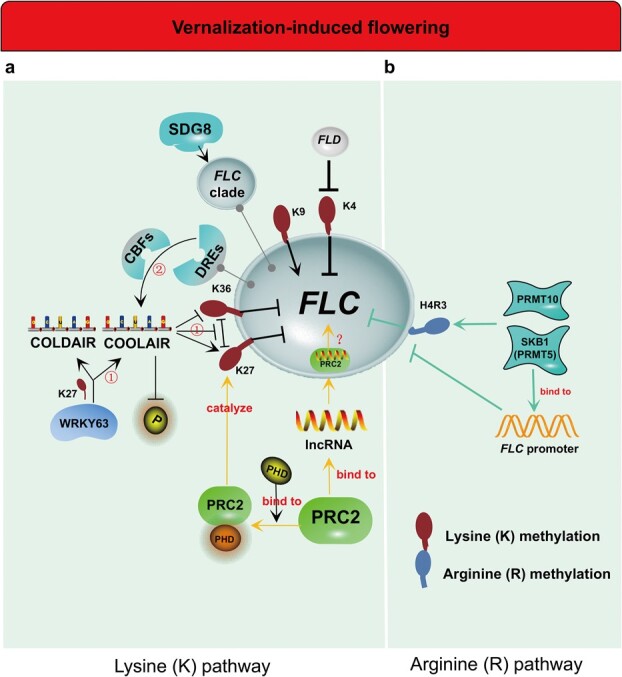
Histone methylation in vernalization-induced flowering. At the center is *FLOWERING LOCUS C* (*FLC*)*.***a** Lysine (K) and **b** arginine (R) histone methylation pathways regulate *FLC* expression through the *FLC* promoter, Polycomb Repressive Complex 2 (PRC2), long non-coding RNA (lncRNA), *COOLAIR* (an lncRNA), *FLOWERING LOCUS D* (*FLD*), Shk1 binding protein 1 (SKB1) [functioning as protein arginine methyltransferase 5 (PRMT5)] and PRMT10. **a** Methylation of H3K4/36 activates *FLC* expression, while the methylation of H3K27/9 has the opposite effect, repressing *FLC* expression and promoting vernalization-­induced flowering. There are two controversial pathways of *COOLAIR*: (i) *COOLAIR* directly represses *FLC* by the increase in H3K36me3 and decrease in H3K27me3 at the *FLC* locus or is induced by WRKY63 to indirectly repress *FLC* (H3K27me3 reduction occurs), so as to participate in vernalization-induced flowering; (ii) CRT/DRE-binding factors (CBFs) bind to the CRT/DRE of *FLC*, resulting in an increase in *COOLAIR*; CBF mutation causes a serious defect in *COOLAIR*, but shows almost normal vernalization. There are two controversial pathways related to whether *COOLAIR* is a necessity for *FLC* repression during vernalization. SDG8/EFS, a histone lysine methyltransferase, functions in the expression of the *FLC* clade. **b** Both SKB1 (PRMT5) and PRMT10 have potential effects on vernalization. Symbol explanations: → indicates promotion; ↔ indicates interaction; $ \dashv $indicates inhibition or silence. The followings of symbol explanations are the same.

The histone methylation sites that have been identified occur chiefly on the residues of lysine and arginine [21], which covalently modify H3 and H4 histones to affect vernalization. For lysine (Fig. 1a), *FLC*, as the main locus of histone modifications, converges various signals of flowering. In *Arabidopsis*, Polycomb Repressive Complex 2 (PRC2), required for vernalization, can methylate vernalized genes near the histone region. PRC2 catalyzes histone 3 lysine 27 trimethylation (H3K27me3) to inhibit gene expression (Fig. 1a). In addition, cold (vernalization) induces plant homologous domain (PHD)-PRC2, a modified PRC2 with PHD proteins (VIN1/3/5, VRN5), to trigger high levels of H3K27me3 and stable epigenetic repression at *FLC* locus [22–24] (Fig. 1a). Interestingly, vernalization makes it possible to hold a steady memory of the long-term cooling effect after rewarming [18], which allows PHD-PRC2 to continue to output H3K27me3 and epigenetic inhibition [25]. Eventually even PHD-PRC2 and H3K27me3 cover the entire *FLC* locus [25, 26]. This is the main reason that *FLC* remains silent in the growth of plants from now on. PRC2, prior to *FLC* silencing by vernalization, binds specifically to long non-coding RNAs (lncRNAs) to form PRC2-lncRNAs, which are recruited to a specific target, i.e. *FLC* [22] (Fig. 1a). Polycomb of PRC2 silences target genes by modifying histone, particularly methylating histone (H3K27me3, H3K36me3, and H3K4me3), and thus polycomb is widely involved in pivotal life processes such as development, proliferation, and differentiation. *COOLAIR* is transcribed antisense from the *FLC* locus induced by cold. Antisense RNA is a specific type of non-coding RNA (ncRNA) modulating genetic activity in cells at multiple levels [27]. Growing evidence reveals the functionality and biological relevance of ncRNAs. According to its function, plant ncRNA is divided into two types: regulatory and structural ncRNAs. The regulatory ncRNAs mainly include lncRNA, circular RNA (circRNA), and small RNAs (sRNAs) containing microRNA (miRNA), short interfering RNA (siRNA), and PIWI-interacting RNA (piRNA); the structural ncRNAs includes ribosomal RNA (rRNA), transfer RNA (tRNA), small nuclear RNA (snRNA), and small nucleolar RNA (snoRNA) and the like [28–30]. The lncRNA *COOLAIR*, which notably causes polycomb silencing, has been found to boost transcriptional shutdown of *FLC* in *Arabidopsis* [5, 22, 26, 31, 32]. Further, at the *FLC* locus, *COOLAIR*-mediated demethylation of H3K36me3 and trimethylation of H3K27 coordinate to silence *FLC*, which in parallel regulate blossoming during vernalization [5, 22, 26] (Fig. 1a). In addition, plants with a mutation of the group-III WRKY transcription factor WRKY63 exhibit an early-flowering phenotype [31, 33]. Interestingly, WRKY63 directly activates *FLC* under non-vernalization, but indirectly represses *FLC* by inducing two lncRNAs, *COOLAIR* and *COLDAIR*, during vernalization, and in this process the reduction of H3K27me3 occurs [31] (Fig. 1a). Another study has shown that *COOLAIR*-mediated *FLC* repression during vernalization is significantly related to cell size [32]. However, some reports showed that *COOLAIR* only participates in *FLC* repression at room temperature, and does not mediate *FLC* regulation during vernalization [34, 35] (Fig. 1a). A later study found that the vernalization-triggered *COOLAIR* mechanism requires C-repeat (CRT)/dehydration-responsive elements (DREs) at the 3′-end of *FLC* and CRT/DRE-binding factors (CBFs) [34]. During vernalization, CBFs can bind to the CRT/DRE of *FLC in vitro*/*vivo*, resulting in an increase in *COOLAIR* transcripts, while mutation of CBFs leads to a serious *COOLAIR* defect, but shows an almost normal vernalization response [34] (Fig. 1a). Hence, *COOLAIR* is probably not a necessity for vernalization. This is consistent with the study of Helliwell *et al*. [35]. *Arabidopsis* with no increase in antisense transcription during vernalization showed normal *FLC* repression and corresponding H3K27me3 changes [35], indicating that *COOLAIR* is probably not an indispensable part of vernalization-induced *FLC* repression. Moreover, before PRC2 adds H3K27me3 to *FLC*, PRC2 was revealed to play a potential role in the establishment of *FLC* repression [35]. However, some studies in *Arabidopsis* argued that the mechanism of *FLC* silencing leading to flowering is the synergistic effect of PRC2 and *FLOWERING LOCUS D* (*FLD*, the floral accelerator via repressing *FLC*) complex by upregulation of H3K27me3 and downregulation of H3K4me2 [36, 37] (Fig. 1a). In addition to the above *FLC* pathway, lysine methylation involved in vernalization-induced flowering may also act in the following pathways. SDG8/EFS (SET Domain Group 8/Early Flowering in Short days), a histone lysine methyltransferase, is required for gene expression of *FLC* clade and functions in delaying vernalization-induced flowering in *Arabidopsis* [38] (Table 1). Additionally, as an identified H3K27me3 reader, BAH domain-containing transcriptional regulator 1 [BDT1 (AT4G11560/AIPP3)] modulates *Arabidopsis* flowering time [39] (Table 1). However, it remains to be seen whether BDT1 is a player in vernalization. The methylation of H3K4/36 activates *FLC* expression and suppresses vernalization-induced flowering, while the methylation of H3K27/9 has the opposite effect, repressing *FLC* expression and promoting vernalization­-induced flowering (Fig. 1a). In winter wheat, the upregulated expression of vernalization-responsive genes *VERNALIZATION 1* (*TaVRN1*) and *FLOWERING LOCUS T*-*like 1* (*TaFT1*) in *Triticum aestivum* by vernalization is linked to the increased level of H3K4me3 [40], suggesting that the floral transition is probably related to histone methylation. During vernalization, VRN1 is activated by a decrease in H3K27 methylation and by an increase in H3K4 methylation to repress VRN2, and thereby promote *Agrostis stolonifera* flowering [41]. In a collaborative study of vernalization and photoperiod, histone lysine (K)-mediated methylation changes in VRN genes probably bypassed the vernalization requirement for flowering to boost flowering, in which miR39 is involved [41]. Transgenic plants overexpressing miR396 show advanced *A. stolonifera* flowering via enhancing expressions of VRN1 and VRN3, which are accompanied by alteration of H3K4/27 methylation [41]. The are two possibilities for the bypass: one is that histone methylation initiates pathways other than vernalization and induces flowering, and the other is that histone methylation substitutes (partially) for vernalization to induce flowering.

**Table 1:** Methylation modifiers functionally characterizes in flowering.

	**Name**	**Accession number**	**Molecular function**	**Biological role**	**Plant**	**Reference**
Histone methylation	SDG8/EFS	AT1G77300	Histone lysine methyltransferase	Vernalization-induced flowering	*Arabidopsis*	[38]
	BDT1/AIPP3	AT4G11560	H3K27me3 reader	Flowering	*Arabidopsis*	[39]
	AtPRMT5	NP_194841	Histone arginine methyltransferase	Vernalization-induced flowering	*Arabidopsis*	[51]
	AtPRMT10	NP_563720	Histone arginine methyltransferase	Vernalization-induced flowering	*Arabidopsis*	[52]
	SKB1/PRMT5	AT4G31120	H4R3 symmetric dimethyltransferase	Flowering	*Arabidopsis*	[53]
	AtPRMT6	AT3G20020	Histone arginine methyltransferase	Photoperiodic flowering	*Arabidopsis*	[137]
	SDG723/OsTrx1	LOC_Os09g04890	H3K4/36 methyltransferase	Flowering	*Oryza sativa* spp. *japonica*	[138]
	SDG724	LOC_Os09gl3740	H3K36 methyltransferase	Flowering	*O. sativa* ssp. *indica*	[139, 143]
	SDG725	NM_106379	H3K36 methyltransferase	Hormone regulatory, gene activation, flowering	*O. sativa* spp. *japonica*	[140, 141, 143]
	SDG718/711	XP_015630972.1/ XP_015644234.1	H3K27 methyltransferase	Photoperiodic flowering	*O. sativa* spp. *japonica*	[142]
	SDG708	XP_015633505.1	H3K36 methyltransferase	Photoperiodic flowering	*O. sativa* spp. *japonica*	[143]
	SDG712	XP_015623394.1	H3K9 methyltransferase	Photoperiodic flowering	*O. sativa* spp. *japonica*	[143]
	SDG25	NP_001318731.1	H3K36 methyltransferase	Vernalization-induced flowering, photoperiodic flowering	*O. sativa* spp. *japonica*	[144, 145]
	SDG26	NP_177797.2	H3K4/36 methyltransferase	Vernalization-induced flowering, photoperiodic flowering	*O. sativa* spp. *japonica*	[145]
Histone demethylation	JMJ18	AT1G30810	H3K4 demethylase	Photoperiodic flowering	*Arabidopsis*	[110]
	JMJ27	AT4G00990	H3K9 demethylase	Photoperiodic flowering	*Arabidopsis*	[127]
	JMJ14	AT4G20400	H3K4 demethylase	Flowering	*Arabidopsis*	[131, 132]
	JMJ12/REF6	AY664499	H3K27 demethylase	Flowering	*Arabidopsis*	[136]
	JMJ11/ELF6	AY664500	H3K27 demethylase	Flowering	*Arabidopsis*	[136]
DNA methylation	LoCMT	Contig64861	DNA methyltransferase	Vernalization-induced flowering	Oriental hybrid lily ‘Sorbonne’	[98]
RNA methylation	FIONA1	AT2G21070	RNA *N^6^*-methyladenosine methyltransferase	Photoperiodic flowering	*Arabidopsis*	[151, 153, 154]
	METTL4	AT1G19340	*N^6^*-adenosine methyltransferase	Vernalization-induced flowering, photoperiodic flowering	*Arabidopsis*	[155]
Polycomb silencing	OsiEZ1/SDG718	LOC_Os03g19480	H3K27 methyltransferase	Flowering	*O. sativa* spp. *japonica*	[142]
	OsCLF/SDG711	LOC_Os06gl6390	H3K27 methyltransferase	Flowering	*O. sativa* spp. *japonica*	[142]

These genes have been submitted to the DDBJ/EMBL/GenBank/TAIR databases see in corresponding literatures.

For arginine (Fig. 1b), the methylation of arginine plays an essential regulatory role in PTM [42]. Arginine (R) is distributed in nuclear and cytoplasmic proteins, and controls the processes of chromatin remodeling, gene transcription, cell proliferation, and differentiation in animals and plants [43–46]. Chromosome recombination and RNA transcription [47] in plants are regulated by the catalytic action of protein arginine methyltransferases (PRMTs). The key part is arginine methylation in the tail of core histones. A series of complex PTMs, including methylation, occur at the N- and C-termini of histones [46]. A methyl group at *S*-adenosyl-l-methionine (AdoMet or SAM) is transferred to the end of guanidinium nitrogens of arginine residues via PRMTs. This removal process generates monomethyl-arginine, symmetric dimethyl-arginine, and asymmetric dimethyl-arginine [46]. There have been preliminary studies of the roles of arginine in vernalization-induced flowering. The pre-mRNAs of plant serine/arginine-rich (SR) protein is widely selectively spliced, which greatly increases the transcriptional complexity. SRs are essential for plant development. Overexpression of *atSRp30* involves pre-mRNA splicing and growth in plants, leading to a late-flowering phenotype after vernalization treatment [48]. Histone arginine methylation is a requisite for vernalization-induced epigenetic silencing of *FLC* [49]. PRMT, an evolutionarily conserved enzyme family, has been illustrated in a previous review on the regulation of flowering time, vegetative growth, the physiological cycle, and the response to high medium salinity and abscisic acid (ABA) [50]. Mutation of AtPRMT5 in *FLC* chromatin leads to pleiotropic phenotypes, including low sensitivity to vernalization, delayed growth and postponed flowering [51]. Shk1 binding protein 1 (SKB1, also known as PRMT5) and AtPRMT10 influence *Arabidopsis* flowering [52, 53] (Table 1). AtPRMT10 has self-methylation activity and its mutation represses *FLC* expression, thereby resulting in late flowering [52]. In contrast, SKB1 catalases the formation of histone H4R3 symmetric dimethylation (H4R3sme2) in *FLC* chromatin, and stimulates blossoming by competitive binding with the *FLC* promoter [53] (Fig. 1b). The late-flowering phenotype of *skb1–1flc-3* is inhibited, verifying that the early flowering function of SKB1 is realized by repressing *FLC* [53]. Interestingly, the reduction of H4R3sme2 due to the mutation of SKB1 does not affect the asymmetric H4R3me2. The SKB1 mutation just upregulates *FLC* expression and triggers late flowering under short days (SDs) or long days (LDs), whereas this late-flowering effect may be reversed by vernalization. Fascinatingly, crosstalk between histone arginine and lysine methylation during vernalization-induced flowering has been verified. During vernalization, *atprmt5* mutant plants cannot acquire the *FLC* inhibitor H3K27me3 [54]. Vernalization-mediated methylation of H3K27/9 at the *FLC* locus requires the methylation of H4R3 in *Arabidopsis* [49]. Accordingly, SKB1/PRMT5-mediated H4R3sme2 is a new histone marker that is required for repressive expression of *FLC* and regulation of flowering time. Another study has further confirmed this. Single mutation of AtPRMT4a/AtPRMT4b does not lead to delayed flowering, but double mutation causes late flowering during vernalization due to hypomethylation of H3R17 and upregulation of *FLC* expression [55]. Four PRMTs (AtPRMT5/10 and AtPRMT4a/4b) are involved in vernalization-induced flowering based on the regulation of *FLC* expression (Fig. 1b). Moreover, vernalization is initiated by VIN3 and then maintained by VRN1, VRN2, LIKE-HETEROCHROMATIN PROTEIN1 (LHP1), and PRMT5. PRMTs seem to be vital markers involved in vernalization-induced flowering. Arginine methylation at the *FLC* locus may be essential for the stability of the vernalized state.

### DNA methylation changes before and after *FLC* reactivation, the substitutability of DNA methylation for vernalization, and the heritability of methylation memory

In *Arabidopsis*, it was reported that a transient and slight decrease in DNA methylation might be related to vernalization-induced flowering [56]. In plants, the main consequences of DNA methylation are CG, CHG, and CHH. Guo *et al*. classified the process of floral transition into three phases through comparative transcriptome analysis between OB (*Rosa chinensis ‘*Old Blush’) and GIG (*Rosa odorata* var. *gigantea*): vegetative meristem (VM), prefloral meristem (TM), and floral meristem (FM) [6]. Then they found that some differentially expressed genes (DEGs) relevant to DNA methylation in VM-GIG and TM-GIG are primarily engaged in vernalization. Methyltransferase changes the level of DNA methylation and then affects flowering. Some genes and proteins with catalytic activity may also affect plant flowering during vernalization, such as DECREASED IN DNA METHYLATION 1 (DDM1) [57]. The expression of *FLC* is suppressed by vernalization, and is suppressed in plants with DNA hypomethylation. *FLC* is associated with many flowering-relevant signals in vernalization-induced flowering.

**Figure 2:**
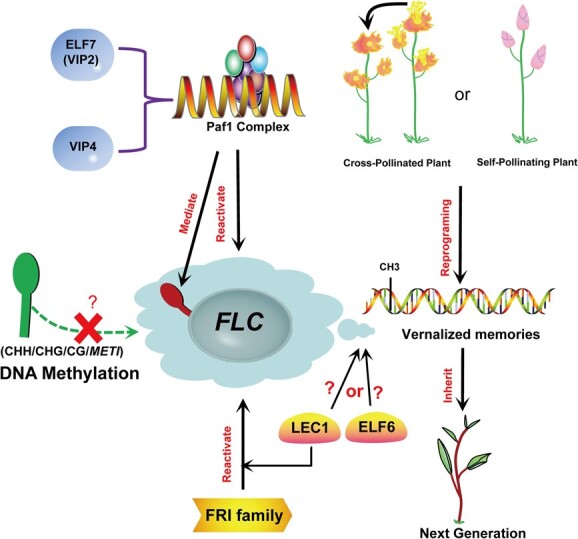
Schematic overview of the effect of DNA methylation on *FLC* reactivation. Yeast RNA polymerase II-associated factor 1 (Paf1) (including VIP2/4) and the FRIGIDA (FRI) family are required for *FLC* reactivation. Apart from perennials, vernalization memory should be reprogrammed at the end of the life cycle, such as the reactivation of *FLC* expression. The vernalization state/memory of parents in *FLC* may be reset by ELF6 or LEAFY COTYLEDON 1 (LEC1), and LEC1 may be involved in the reactivation of *FLC* by FRI. DNA methylation may have no effect on *FLC* reactivation.

Apart from perennials, vernalization every generation is necessary for blossoming, so the cell memory of the vernalization experience should be reprogrammed when the life cycle is approaching the terminal stage [57]. This means that in the memory system, plants need to reactivate the cleared *FLC* expression before the developmental completion of seeds or upon fertilization, and then guarantee and maintain appropriate floral behaviors in each round of flowering [58] (Fig. 2). Notably, a pivotal example of plant epigenetic (methylation) reprogramming is the resetting of *FLC* expression in *Arabidopsis* [59]. A study has shown that methylation with vernalization memory may not be effectively erased due to the mutation of the jumonji-domain-containing protein ELF6, so part of the *FLC* expression may be retained and unite the methylation memory to be inherited by the next generations (with early flowering phonotype) [59]. However, Tao *et al*. demonstrated that the vernalization state/memory of *Arabidopsis* parents in *FLC* is mainly reset by a factor other than ELF6, namely the seed-specific transcription factor LEAFY COTYLEDON1 (LEC1), the main regulator of embryogenesis [60] (Fig. 2). LEC1 boosts the initial establishment of the *FLC* chromatin state and activates *FLC* re-expression in the pro-embryo, thus reversing the epigenetic silencing state (marked by H3K27me3) inherited from gametes [60]. A review from Niu and He shows that three *LEC* genes function in resetting the vernalization memory of parents during early embryogenesis in *Arabidopsis* [61]. Shortly after fertilization, *LEC1* reactivates *FLC* as a pioneer, and completely activates *FLC* with the cooperation of *LEC2* and *FUSCA3* (*FUS3*). Meanwhile, *LEC2* and *FUS3* [recruiting FRIGIDA (FRI), a complex required for *FLC* reactivation] gradually increase in the cold memory element (CME) region, while the level of homologous B3 protein VP1/ABI3-like (VAL) protein [recruiting Polycomb-group (PcG) proteins, like PRC2, a H3K27 methyltransferase complex] gradually decreases. This leads to the destruction of the original epigenetic silencing state of embryonic *FLC*, an adaptive means for plants to prevent precocious flowering before cold exposure in winter. Yeast RNA polymerase II-associated factor 1 (Paf1) is a necessity for high expression and reprogramming of *FLC* [62]. *EARLY FLOWERING 7* (*ELF7*) and *VERNALIZATION INDEPENDENCE4* (*VIP4*), *Arabidopsis* homologous genes of members of the Paf1 complex, are involved in vernalization. Mutation of the two genes causes severe defects in *FLC* reactivation [58] (Fig. 2), which means they are essential to the reactivation. Histone methylation of *FLC* chromatin is mediated by the Paf1 complex and interacts with the vernalization-responsive process [38, 62, 63]. Unlike histone methylation, effectors referred to the DNA methylation pathway are seemingly not involved in *FLC* regulation during reproduction. Thus, we will focus on the role of DNA methylation in *FLC* reprogramming. DNA methylation has no effect on gametogenesis-specific *FLC* repression, and *METHYLTRANSFERASE1* (*MET1*) is not involved in *FLC* reactivation [58]. On the contrary, the low CG DNA methylation in the sporophyte generates *FLC* inhibition, but the DNA methylation at the *FLC* site does not change. In addition, *FLC* activation requires the FRI family, which is a determinant of the vernalization-requiring and winter-annual habit in *Arabidopsis* [64]. *FRI*, as the upstream activator of *FLC*, is indispensable to the complete reactivation of high-level *FLC* during embryogenesis (Fig. 2). However, no matter how active the FRI complex is, DNA methylation still does not participate in the *FLC* reactivation in the reproductive stage. Finnegan *et al*. also showed that DNA methylation does not seem to be involved in the resetting of *FLC* expression before vernalization in *Arabidopsis* [65]. All the above suggests that DNA methylation may really have no effect on *FLC* reactivation. The specific molecular process remains to be explored.

**Figure 3:**
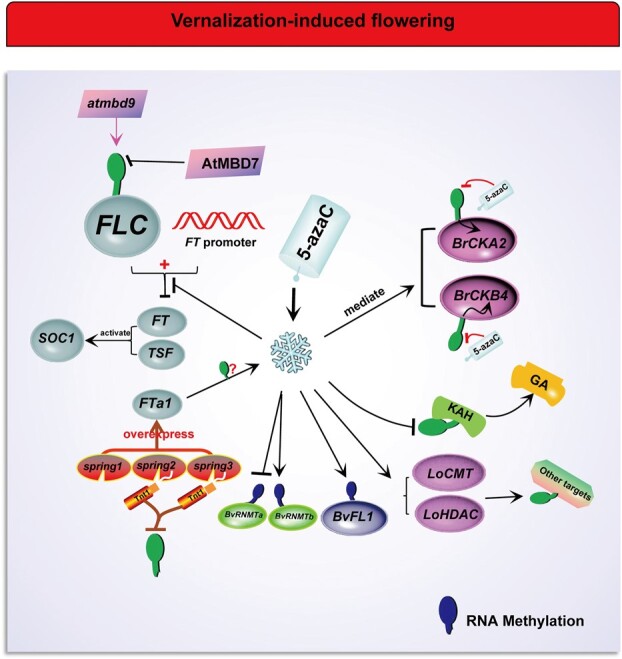
Effects of DNA and RNA methylation on vernalization-induced flowering after *FLC* reactivation. Under the synergistic or independent action of vernalization signals and photoperiod signals, DNA methylation may directly/indirectly affect the expression of the central flowering repressor *FLC* and its homologous gene *FLF*, vernalized genes (*BrCKA2* and *BrCKB4*), mobile florigen *FLOWERING LOCUS T* (*FT*) and its homologous genes *TWIN SISTER OF FT* (*TSF*) and *FTa1* (overexpressed in *spring*), flowering integrator *SUPPRESSOR OF OVEREXPRESSION OF CO1* (*SOC1*) and clock gene *BrCCA1*. AtMBD7 is necessary for active demethylation. AtMBD9-mediated methylation at *FLC* promotes early flowering. 5-Azacytidine (5-azaC) promotes vernalization-induced flowering, which may be related to the GA biosynthetic enzyme gene KAH with apex specificity. In addition, vernalization induces upregulated expression of methylated genes *LoCMT* and *LoHDAC*. RNA methylation may regulate flowering through RNA methyltransferase (RNMT) and the *FLC* homologous gene *FLOWERING LOCUS 1* (*FL1*) in vernalized sugar beet. Snowflakes represent prolonged cold (vernalization) and the following snowflakes represent the same.

Nevertheless, when *FLC* is reactivated, DNA methylation plays an important role in vernalization-induced flowering (Fig. 3). In *Arabidopsis*, a study has shown that *FLC* is downregulated by DNA demethylation, which may occur through a separate vernalization-independent pathway [65]. However, some opposite arguments are that *FLC* or *FLC* regulators are actually controlled by DNA methylation during vernalization [5] and that the *FLC* locus is epigenetically silenced by vernalization [59]. In addition, *FLOWERING LOCUS F* (*FLF*, a repressor of flowering), the homologous gene of *FLC*, is suppressed by vernalization and by a decrease in genomic DNA methylation in *Arabidopsis* [66]. Another study showed that a reduced level of DNA methylation represses *FLC* expression and then facilitates flowering in *Perilla frutescens* (a vernalization-requiring short-day plant) and *Silene armeria* (a non-vernalization-requiring long-day plant) under a non-inductive photoperiod [67], suggesting an efficient effect of the reduced DNA methylation on *FLC* repression during vernalization. Therefore, more experiments are needed to determine whether DNA (de)methylation is involved in the process called vernalization silencing *FLC* after *FLC* reactivation. Actually, there are increasing clues to the involvement of DNA (de)methylation in vernalization-induced flowering. A recent study supports this argument [68]. In the early vernalization stage, an increase in genomic DNA methylation was observed in orchardgrass; mCHH occurring in the promoter region of vernalization-related genes contributes to the upregulation of these genes in this stage; both exogenous application of the DNA methylation accelerator methyl triflate (MTFMS) and overexpression of the RNA-directed DNA methylation (RdDM)-related gene *NUCLEAR POLY* (*A*) *POLYMERASE* (*DgPAPS4*) elevate the DNA methylation level, thus ultimately advancing vernalization-induced flowering [68]. These results indicate the important role of DNA hypermethylation in vernalization-induced flowering. DNA hypermethylation induced by vernalization is one of the inducements for the initiation and development of the floral primordium during vernalization.

*FT*, a vital mobile florigen, can take advantage of vernalization to break *FLC* entrapment and facilitate flowering. Before vernalization, *FT* is restrained by interaction with *FLC* and the *FT* promoter. When vernalization is initiated, vernalization begins to relieve the inhibited transcription of flowering genes. For example, *FT* shows to almost completely overcome for the flowering delay effect of *FLC* via some signals acting in phloem, and subsequently promotes early flowering in *Arabidopsis* [69–71]. Some researchers thought that *FT* and *TWIN SISTER OF FT* (*TSF*) bypasses the flowering barrier produced by *FLC* by activating *SOC1* expression in *Arabidopsis* [72]. *FTa1* is identified as an important flowering time gene in *Medicago truncatula* and the only *FT* gene upregulated by long days and vernalization simultaneously [73]. The synergistic effect of vernalization and long-day signal in *M. truncatula* indicates that *FTa1* mutation delays flowering, while overexpression of *FTa1* accelerates flowering violently [73, 74] (Fig. 3). However, during vernalization, *FT* may not regulate flowering through changes in DNA methylation. *Medicago truncatula spring2* and *3* mutants overexpressing *FTa1* flower early [75] (Fig. 3). *Tnt1* retroelement marking at the *FTa1* locus eliminates the vernalization requirement. The retroelement is usually correlated with block of elevated DNA methylation. Analysis using mock digestions (using McrBC, a methylation-dependent restriction enzyme) shows that no changes in DNA methylation occur at the *FTa1* locus in *spring* mutants [75] (Fig. 3). This means that DNA methylation does not seem to be directly involved in vernalization-induced flowering promoted by *FT*. However, a recent study in orchardgrass (*Dactylis glomerata*) indicates that *FT* may be associated with the increase in DNA methylation involved in vernalization pathways [68]. It was shown that the DNA methylation accelerator MTFMS can promote the expression of vernalization-related genes, including *FT*, *CURLY LEAF* (*CLF*), *FERTILIZATION INDEPENDENT ENDOSPERM* (*FIE*), *MULTICOPY SUPPRESSOR OF IRA* (*MSI*), *VIN3*, *VIN3 LIKE 1* (*VIL1*), *SWINGER* (*SWN*), *VRN1*, and *AGL20* [68]. However, many details about the involvement of *FT* in the regulation of DNA methylation-mediated flowering during vernalization are still unknown.

Intriguingly, other studies indicate that vernalization-mediated flowering promotion is mediated by DNA demethylation [56, 76]. Vernalization probably induces flowering by DNA demethylation at specific sites in the genome. For example, a DNA-methylation inhibitor, 5-azacytidine (5-azaC), may regulate floral regulatory networks to accelerate flowering of multiple species. Some reports revealed the induction of flowering by 5-azaC in flax (*Linum usitatissimum*) [77–79] and *Pharbitis nil* [80]. DNA hypomethylation mediated by 5-azaC elevates the flowering percentage on explants from young, vernalized roots of chicory (*Cichorium intybus*) [81]. In late-flowering ecotypes and mutants, vernalization-requiring *Arabidopsis* treated with 5-azaC can boost flowering, which demonstrates that demethylation may mediate vernalization-induced flowering through the control of one or more genes essential for flowering induction [76]. This control probably includes a step related to a GA biosynthetic enzyme gene with apex specificity (Fig. 3). GA is a phytohormone required for floral transition, and kaurenoic acid hydroxylase (KAH) is the rate-limiting enzyme in its biosynthesis [82]. Interestingly, vernalization leads to demethylation of the KAH gene and subsequent transcriptional activation, resulting in the increased production of GA essential for floral transition of *Arabidopsis* [82] (Fig. 3). DNA demethylation also induces vernalization and flowering of *Arabidopsis* via the repression of *FLC*, the central repressor of the flowering response [65]. Moreover, the vernalization-mediated genes *BrCKA2* (casein kinase II α-subunit) and *BrCKB4* (casein kinase II β-subunit) show increased expression and DNA demethylation in vernalized *Brassica rapa* that is deficient in DNA methylation through silencing *MET1* or applying 5-azaC [83]. By silencing *BrCKA2* and *BrCKB4*, the vernalized *B. rapa* delays flowering, suggesting that *BrCKA2* and *BrCKB4* may be positive regulators of flowering, and there is a potential positive relationship between DNA demethylation and flowering during vernalization [83] (Fig. 3). However, in *Arabidopsis*, although the new function of the casein kinase 2 (CK2) α subunit in flowering has been validated, it does not seem to be involved in vernalization-mediated flowering [84–86]. The questions of whether CK2 is involved in vernalization-induced flowering and whether the corresponding DNA methylation occurs still need additional exploration. DNA demethylation by 5-azaC or methyltransferase mutation promotes vernalization-induced flowering, which means that maybe we can replace the effect of cold (vernalization) on flowering via demethylating. We propose a hypothesis that DNA demethylation mediated by externally ingested 5-azaC may replace vernalization by inducing early flowering in vernalization-requiring lines or otherwise provide an avenue to manipulate flowering time. This hypothesis has been partly confirmed. In vernalized winter wheat, reagent 5-azaC and γ rays can partially substitute for vernalization to boost flowering [87]. In vernalized *Arabidopsis* and *C. intybus*, 5-azaC and antisense RNA can partially substitute for the vernalization requirement by repressing methylase activity [88]. A recent study has also shown the possible substitution of 5-azaC for vernalization to accelerate flowering in Iranian *Anemone* accessions [89]. They explored the possibility of pretreatment with GA3 and 5-azaC substituting for cold storage (vernalization) in Iranian *Anemone* accessions and found that both 5-azaC and GA3 treatments advance *A. biflora* flowering under non-vernalized conditions [89]. These results suggest that the substitutability of DNA methylation for the cold effect (vernalization) exists. However, it is also controversial that 5-azaC, as a demethylating reagent, is also a universal inhibitor of transcription, and thereby the promotion of flowering by 5-azaC might be caused by effects other than DNA demethylation. Because of this controversy, some studies used *Arabidopsis* plants in which the DNA methylation level is decreased by antisense *METI* transgenes or *DDM1* mutation [76, 90, 91]. DNA-methylation-deficient plants bloom early without vernalization, suggesting that demethylation is enough to prompt early flowering. Therefore, although it cannot be asserted, 5-azaC is likely to use the DNA demethylation function in promoting flowering. Additionally, demethylation by antisense *MET1* has been proved to have a superimposing effect on promotion of flowering by low temperature in winter wheat or *Arabidopsis* [76]. The above shows that downregulated DNA methylation (demethylation) is a potential substitute for vernalization to promote flowering.

However, upregulated DNA methylation levels may also contribute to vernalization. Methyl-CpG-binding domain (MBD) protein, a vital *trans*-acting factor, functions in specially recognizing/interpreting methylated DNA [92]. MBD interacts with chromatin remodeling protein to silence genes. In *Arabidopsis*, by mutating AtMBD8 of C24 (vernalization-responsive accession), *atmbd8-1* displays late flowering under long days/short days, downregulated expression of *FT1* and *SOC1*, and the same response to vernalization as the control [93]. These results suggest that AtMBD8 is a novel flowering promoter, but may not be involved in vernalization-induced flowering. However, the mutation of AtMBD9 induces DNA methylation at the *FLC* locus in the vernalization pathway and leads to *Arabidopsis* early flowering [94]. MBD protein has been proved to be associated with active DNA methylation (Fig. 3). For example, AtMBD7 is necessary for active demethylation and anti-silencing of high-density DNA methylation [95]. Hence, MBD is likely to serve as a reminder of changes in DNA methylation during vernalization. MBD has exhibited similar functions in other species. In wheat with vernalization treatment, TaMBD6 homologs are differentially expressed in developing wheat [96]. Among them, the expression of TaMBD6_B and TaMBD6_D is upregulated, while the expression of TaMBD6_A is very weak, indicating that TaMBD6 is induced by prolonged chilling and may be involved in the vernalization-induced transition to flowering [96]. A recent study in *Chrysanthemum lavandulifolium* found that MBD protein may recognize flowering control genes regulated by DNA methylation in the vernalization pathway [97]. Of the 89 candidate genes identified by MBD sequencing, 49 genes exhibited changes in DNA methylation status during flowering induction [97]. Among them, *VIP2* in the vernalization pathway, *FCA* and *REF6* in the autonomic pathway, *ZTL* in the photoperiod pathway, and *GAI* in the GA pathway were identified as flowering control genes highly likely to be regulated by DNA methylation [97]. Moreover, the expressions of two DNA-methylated genes of lily, *LoCMT* (CMT-type DNA methyltransferase genes) and *LoHDAC*, are promoted by vernalization [98] (Fig. 3). The gene expression of *LoCMT* and *LoHDAC* increases with increasing vernalization time [98]. In other words, increased DNA methylation may also have a positive regulatory effect on vernalization. Above all, DNA methylation and demethylation may all have a positive regulatory effect on vernalization-induced flowering. The reason for this contradiction may relate to the fact that flowering behavior is a combined action of various signals in and out of plants, or the different methylation status of different flowering-related genes. In addition, long days are accepted as the most important stimulus correlated to flowering, so interaction of long days with vernalization and methylation and other factors cannot be ignored in the process of flowering induction. Furthermore, the functional scope of 5-azaC as a general transcriptional inhibitor is uncertain, which may require more rigorous verification. After all, little is known about which (de)methylation pathway is specifically affected by 5-azaC in plants. Further studies, which take into account the contradiction mentioned above, will need to be undertaken.

### Latest progress of RNA methylation in the vernalization pathway

Vernalization induces flowering mainly through DNA demethylation and histone modification, which are the results of epigenetic regulation [99]. Vernalization-mediated silencing/promotion of floral regulators probably occurs through histone modifications and DNA methylation. RNA methylation (e.g. m^5^C in RNA) was discovered a long time ago, but its mechanism in some biological processes (e.g. flowering) is still equivocal [100]. Perhaps its role might lie in the effectiveness of mRNA translation and stability. Very few studies have been devoted to the role of RNA methylation in vernalization-induced flowering. In the study of vernalized sugar beet (*Beta vulgaris altissima*, a long-day plant) [101], like phenolic compounds, RNA methylation also plays a role in floral transition. In identified differentially methylated regions (DMRs), two *RNA METHYLCYTOSINE TRANSFERASE* (*RNMT*, DMRs related to bolting) sequences, *BvRNMTa* and *BvRNMTb*, were identified during cold exposure (vernalization) and/or between genotypes [101]. These genotypes are mainly divided into two categories: a sensitive early bolting genotype (S) and a resistant late bolting genotype (R). It is generally acknowledged that sugar beet can bolt without flowering, but seldom flowers without bolting [102], indicating a tight link between bolting and flowering. Contrary to one of the *RNMT* sequences, the other exhibits hypermethylation of the gene body and its expression is activated by cold exposure (Fig. 3). The mRNA methylation of floral suppressor *FLOWERING LOCUS 1* gene including *BvFL1* occurs in the bolting-resistant genotype under cold exposure [101] (Fig. 3). *BvFL1*, an *FLC*-like and vernalized gene, functions as a flowering repressor like *FLC* and is downregulated by vernalization [103]. Methyl RNA immunoprecipitation (MERIP) ensured *BvFL1* could be involved in the RNA methylation pathway; *BvRNMTa* and *BvRNMTb* show CG, CHG, and CHH methylation under cold exposure; the former gene shows hypomethylation and expression silencing, while the latter shows hypermethylation and expression activating (Fig. 3); the S genotype has a higher total RNA methylation level than the R genotype [101]. These results suggest that *RNMT* (RNA methylation) plays a role in bolting and flowering under cold treatment. Consistent with the study on the *Arabidopsis rnmt* mutant, the RNA methylation level decreases in *rnmt* vernalized sugar beet. Surprisingly, under short days cold treatment can still promote bolting and flowering in the *rnmt* mutant. However, an *Arabidopsis rnmt* mutant experiment demonstrated that *RNMT* does not seem to be necessary for stem bolting and flower formation [101]. Overall, *rnmt* mutants show the phenomenon of early bolting/flowering, suggesting that vernalization can accelerate the bolting and flowering process through *RNMT*. RNA methylation may promote or delay bolting and flowering by regulating vernalization at the RNA level. But more information is still required about RNA methylation for vernalization; this may be a more open question than histone and DNA methylation.

Generally, methylation interacts with temperature (vernalization) to regulate blossoming through a variety of regulatory factors. Among the three major methylation modes, vernalization­-dependent plants may regulate flowering behaviors mainly through histone modification and DNA methylation. In plants, histone and DNA methylation may influence vernalization-induced flowering mainly around *FLC*, followed by other flowering-related genes, sRNAs, phytohormones, and the MBD. Of course, a broader and more complex mechanism of RNA methylation may be involved in vernalization-induced flowering, and this needs careful exploration.

## Methylation is involved in photoperiod-regulated flowering

Each plant has evolved a complex mechanism of flowering transition. Photoperiod, light quality, and light intensity, like temperature (vernalization), deeply affect the timing of flowering in plants [76]. A study has found that light quality has a significant effect on plant flowering [104]. In *Arabidopsis*, far-red light and blue light can foster flowering but, inversely, red light may postpone flowering [104]. *CO*, *FT*, *CRYPTOCHROME 2* (*CRY2)*, *FHA*, *GIGANTEA*, and *FWA* are the main regulators in the flowering pathway, and are induced by photoperiod [105–107]. In *Arabidopsis*, light (photoperiod, light quality) and temperature (vernalization) are the external conditions influencing the pivotal floral transition, and they play a role through the *CO*-*FT* and *FLC* pathways, respectively [4, 108]. Light signals measure the time of flowering, and CO is a key link protein used by light signals to regulate flowering time [18, 109]. Thus, CO is an important protein that mediates plant circadian clocks and flowering time. *FLC* is a classical central flowering suppressor. *FLC* can integrate vernalization, autonomy, and other pathways to regulate flowering in a dosage-dependent manner [110]. *FT*, as a component of the photoperiod-induced pathway, orchestrates signals from photoperiod, vernalization, and autonomy to stimulate flowering with increasing day length. *FT*, which is activated by *CO*, integrates photoperiod-dependent and *FLC*-dependent pathways to regulate flowering time by controlling the expression of flowering-specific identity genes [110]. After the integration of flowering signals in the photoperiod pathway and *FLC* signals, the expression of common downstream integrators of flowering, such as *SOC1* and *FT*, is controlled [18, 104, 111]. These expressions are restrained by *FLC*, while the photoperiodic floral regulatory signals mediated by *CO* antagonize *FLC*, and *CO* is responsible for activating the expression of these integrators [18, 104, 111]. Thus, there is an antagonistic relationship between *CO* and *FLC* in the promotion of flowering. Photoperiodic flowering is probably affected by a variety of chromatin modifications in the plant, such as DNA methylation, H3K4 methylation, and RNA methylation, by controlling floral regulatory behaviors of *FLC* and *CO*.

### DNA methylation in photoperiodic flowering and its substitution for photoinduction and the heritability of methylation memory

**Figure 4:**
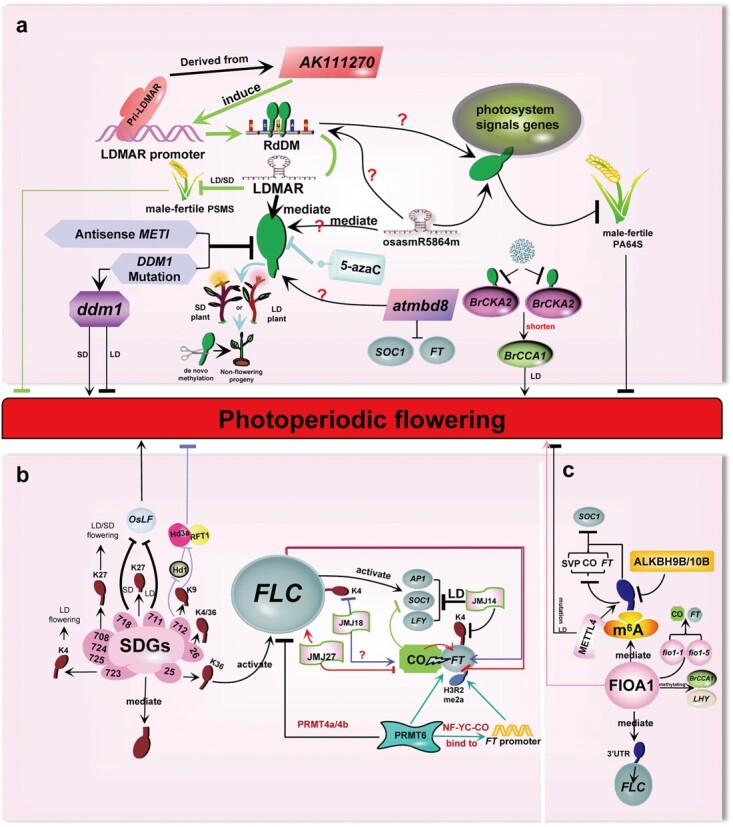
Methylation in photoperiodic flowering. **a** DNA methylation in photoperiod-induced flowering: *METHYLTRANSFERASE* (*METI*), *DECREASED IN DNA METHYLATION1* (*DDM1*), 5-azacytidine (5-azaC) and ncRNA [long-day–specific male-fertility–associated RNA (LDMAR) and osasmR5864m] mediate DNA methylation (RdDM) to affect flowering; methyl-CpG-binding domain (MBD) protein may regulate flowering through methylation; the effect of 5-azaC may be reset in the next generation. **b** Histone methylation in photoperiodic flowering. Two pathways of histone methyltransferase (SDGs and PRMT6) and histone demethylase (JmjC protein) in the photoperiod pathway: a series of SDG-mediated histone lysine methylations participates in photoperiodic flowering by affecting flowering-related regulators *FLC*, *SOC1*, *OsLF* (suppressors of *Hd1*), *Ehd1*, *Hd3a*, and *RFT1*. PRMT6 not only mediates the interaction between the H3R2me2a system and the NF-YC-CO module to dynamically regulate the expression of *FT*, but also cooperates with its homologous protein AtPRMT4a/4b to repress *FLC*, an inhibitor of *FT*, thus promoting photoperiodic flowering. A series of H3K9/4 demethylases such as JmjC domain protein 27 (JMJ27)–JMJ14 play a role in the *FT*-*FLC*-*CO* module and act on upstream and downstream regulatory factors *APETALA 1* (*AP1*), *SOC1*, and *LEAFY* (*LFY*). The long-day (LD) signal and the occurrence of methylation in the photoperiod pathway are coordinated by these JmjC proteins. **c** RNA methylation in photoperiodic flowering: FIONA1 (mediating m^6^A methylation) and METTL4 (with *N^6^*-methylation activity) play respective roles in photoperiodic flowering. FIONA1 participates in the photoperiod pathway by regulating *FLC* methylation and the expression of flowering genes *SOC1*, *SHORT VEGETATIVE PHASE* (*SVP*), *CO*, and *FT*, as well as clock genes *CCA1* and *LATE ELONGATED HYPOCOTYL* (*LHY*). Additionally, the mutation/deletion of METTL4 leads to early flowering under long days.

DNA methylation regulates photoperiodic flowering in plants. MBD is a protein that recognizes methylated DNA. The mutation of AtMBD8 delays photoperiod-induced *Arabidopsis* flowering with the downregulation of *FT* and *SOC1* expression in an *FLC*-independent and *CO*-independent manner [93] (Fig. 4a). AtMBD8, perhaps being a flowering promoter, may regulate photoperiod-induced flowering through DNA methylation [93]. Consequently, MBD may be involved in photoperiod-induced flowering via DNA methylation. In *Arabidopsis*, consistent with antisense *METI*, the mutation of *DDM1* causes DNA demethylation [76, 90] (Fig. 4a). By comparing the flowering time of *ddm1*-homozygous mutants (self-pollination progeny), it was found that *ddm1* mutants flower later under long days [76, 112]. Also, the flowering time in self-pollinated progenies became gradually later generation by generation [76, 112], indicating that DNA demethylation mediated by *DDM1* mutation promotes photoperiodic flowering. This shows that *Arabidopsis* of the ecotype Colombia blossoms mainly through the photoperiod pathway rather than vernalization under long days. Under short days, the progenies of *ddm1* blossom earlier generation by generation, and the effect of promoting flowering is better in *ddm1* with vernalization treatment [76], suggesting that the ecotype depends on vernalization to blossom under short days. This shows that DNA demethylation by *DDM1* mutation inhibits photoperiodic flowering, while it is conducive to promoting vernalization-induced flowering. As one of the main pathways to induce flowering, photoperiod is directly or indirectly related to vernalization. After vernalization is initiated at low temperature, it is not until several weeks or months that plants blossom, and thereby the specific conditions for photoperiod flowering are gradually satisfied during this period [83]. To study the interaction between photoperiod and vernalization in flowering, Duan *et al*. constructed DNA methylation-deficient plants and viral silencing vectors [83]. It was found that vernalization induces DNA demethylation of two subunits of CK2 (*BrCKA2* and *BrCKB4*), which shortens the period of the clock gene *BrCCA1* (an important gene in photoperiod perception) in vernalized *B. rapa* and consequently boosts flowering under long days (Fig. 4). Studies have shown that casein kinases (such as CK1 and CK2) are involved in regulating the flowering time of many plant species, such as *Arabidopsis*, rice, and *B. rapa* [113]. As a consequence, photoperiod and vernalization interact directly through DNA demethylation and synergistically accelerate flowering. In other words, DNA demethylation is able to participate simultaneously in both vernalization- and photoperiod-induced flowering. So far, vernalization and photoperiod are the two pathways that simultaneously regulate flowering time [114, 115]. However, in model plants such as *Arabidopsis*, it is unknown whether DNA methylation participates in both vernalization and photoperiod to regulate flowering. In other species DNA methylation plays the same role in plant flowering. 5-azaC induces the flowering of the long-day plant *S. armeria* and the short-day plant *P. frutescens* var. *crispa* under a non-inducing photoperiod, indicating that flowering gene expression is modulated by DNA methylation [67]. However, the progeny of plants whose flowering is induced by 5-azaC cannot flower under a non-inducing photoperiod because *de novo* methylation occurs in the progeny [67] (Fig. 4a). Regarding heritability, the early flowering phenotype produced by antisense *METI*-mediated demethylation can be passed on to the next generations. DNA demethylation probably regulates photoperiod flowering, but this is independent of the stability of the photoperiod-induced flowering state [116]. Intriguingly, demethylation has also been proved to have a substituting role in photoperiodic flowering. This is the first finding that photoinduction can be replaced by DNA demethylation in *P. frutescens* seeds via 5-azaC treatment [117]. *Perilla frutescens* is an absolute short-day plant and does not require vernalization. Treatment with 5-azaC broke the suppression of *P. frutescens* flowering by long days, and its flowering promotion effect was slightly weaker than that of short-day treatment, which means that 5-azaC can at least partially replace the short-day requirement for flowering in absolute short-day plants [117]. This is similar to our hypothesis stated above in the section ‘DNA methylation changes before and after *FLC* reactivation, the substitutability of DNA methylation for vernalization, and the heritability of methylation memory’ that ‘5-azaC-mediated DNA demethylation may replace vernalization by inducing early flowering in vernalization-requiring lines’, and here DNA demethylation may (partially) replace photoinduction by inducing early flowering in plants that need short days. Not requiring vernalization means that long-day/short-day plants bloom mainly through the photoperiod pathway. Spinach is a long-day plant and does not require vernalization. In another study, co-treatment of 5-azaC and photoinduction greatly promoted spinach flowering compared with the control treatment of photoinduction or 5-azaC, implying that 5-azaC treatment can potentially partly replace photoinduction [118]. These observations suggest that DNA demethylation (induced by 5-azaC) may substitute for photoinduction in photoperiodic flowering. In addition to vernalization- and photoperiod-related genes, DNA demethylation (5-azaC) may also induce/trigger other flowering-related genes during substitution. DNA demethylation (5-azaC) may provide an additional avenue to manipulate flowering time. Therefore, we hypothesize that this is the real reason why DNA demethylation can replace or partially replace the cold effect (vernalization) and photoinduction (photoperiod) to promote plant flowering.

DNA methylation is involved in the flowering of grain crop rice mainly through a mediator: sRNA, such as long-day-specific male-fertility-associated RNA (LDMAR, a kind of lncRNA) and osasmR5864m [119, 120] (Fig. 4a). LDMAR, which controls photoperiod-sensitive male sterility (PSMS) hybrid rice, is necessary for male fertility in rice under long days [121]. The Psi-LDMAR in the LDMAR promoter region probably originates from the AK111270 gene, whose overexpression induces RdDM in the LDMAR promoter and inhibits the expression of LDMAR [119, 122, 123] (Fig. 4a). In other words, the increased methylation of the LDMAR promoter in PSMS rice leads to decreased expression of LDMAR under long days. The decreased LDMAR expression causes male sterility and delays fertility restoration under long or short days. The flowering habits of PSMS rice are affected by RdDM in LDMAR. The male sterility caused by the high methylation may eventually weaken the flowering peak; the average flowering frequency may decline more seriously; and the pre-afternoon flowering ratio may significantly decline. In short, RdDM can lead to flowering obstacles in PSMS rice to some extent. In the photoperiod-thermosensitive male sterile (PTGMS) line PA64S, DNA methylation may also participate in the transformation of sterility and fertility [124]. This transformation is regulated by photoperiod and temperature. Sterility of PA64S can be transformed into fertility under short days and low temperature [125]. Similar to LDMAR in PSMS rice, osasmR5864m, a non-coding and mutant sRNA, is responsible for the sterility–fertility transformation in PA64S, which is closely related to DNA methylation [125] (Fig. 4a). BLAST analysis shows that DNA fragments occur in differential methylations and these differential fragments correlate to photosynthesis, signal transduction, metabolism, and transposon activation [124]. DNA fragments relevant to photosystem signals show hypermethylation. PA64S (S) shows more methylated fragments than PA64S (F) [124]. This means that male sterility may be induced by elevated methylation levels in PA64S rice, which causes poor flowering habit. PSMS and PA64S rice may show male sterility resulting from increased RdDM, which then affects flowering behaviors. In general, DNA hypomethylation seems to be a promoter of flowering and may be a substitute for photoinduction in photoperiodic flowering.

### Histone and RNA methylation in photoperiodic flowering

In contrast to DNA methylation, existing evidence suggests that histone methylation acts on photoperiodic flowering more as a demethylase modifier to regulate flowering (Fig. 4b). Studies show that the photoperiod-mediated *CO*/*FT* module can activate floral transition in chrysanthemum and poplar [6, 126]. *CO*, as a prime flowering regulator, controls the expression of *FT* mRNA through the photoperiod pathway to induce flowering [114]. The expression of the photoperiodic switch gene *CO* is strictly restricted by light and clock genes such as *CCA1* [74]. *FT*, encoding a RAF-kinase inhibitor-like protein, is a flowering activator regulated by vernalization and photoperiod in *Arabidopsis* [114, 115]. *FT* is activated by *CO* in the photoperiod pathway and restrained by *FLC* before vernalization (Fig. 4b). *FT* acts as a component in the photoperiod pathway as well as a floral integrator that integrates the perception of inductive photoperiods and the *FLC*-mediated floral repression signal. JmjC DOMAIN-CONTAINING PROTEIN 27 (JMJ27), one of the H3K9 histone demethylases, was found to be involved in this activation and restraint of *FT* [127] (Fig. 4b). Plants usually respond to environmental stress by regulating the time of flowering, and it has been proved that plant defense signals do exist in the flowering regulation pathway [128–130]. JMJ27 is a protein factor that can regulate both plant defense and flowering time. In flowering, JMJ27 shows positive regulation of the floral repressor *FLC* and negative regulation of the floral activator *CO* [127] (Table 1). The absence of JMJ27 causes early flowering in *Arabidopsis* infected with *Pseudomonas syringae* [127] and thereby JMJ27 may be engaged in photoperiodic flowering. JMJ18 shows demethylase activity of H3K4me2/3 on the induction of photoperiodic flowering in *Arabidopsis* [110] (Table 1). JMJ18 inhibits *FLC* expression by reducing the methylation level of H3K4 in *FLC* chromatin, thus promoting *FT* expression and then stimulating flowering [110] (Fig. 4b). Contrary to JMJ27, JMJ18, which relies on the high expression of functional *FT*, leads to early flowering, while its mutation leads to late flowering. JMJ14, an H3K4 demethylase, is vital for preventing early flowering of *Arabidopsis* in the vegetative stage via the repression of the floral activator *FT* and the floral integrators *AP1*, *SOC1*, and *LFY* in an *FLC*-independent manner under long days [131, 132] (Fig. 4b, Table 1). In *Arabidopsis*, a previous report highlights that JMJ14 functions at the H3K4me3 level in the *FT* transcription initiation region to modulate photoperiodic flowering [133, 134], while others reveal that JMJ14 does not cause any changes in histone methylation levels of the *FT* or *FLC* locus [131, 134]. JMJ30 and JMJ32 directly bind and demethylate H3K27me3 at the *FLC* locus *in vitro*/*in vivo* and modulate *Arabidopsis* flowering under long days [135] (Table 1). Other JmjC group proteins, JMJ11 (ELF6) and JMJ12 (REF6), have a delayed and accelerated effect on photoperiodic flowering in *Arabidopsis*, respectively [136] (Table 1). In addition to these JmjC proteins, histone methyltransferases (HMTases) also function in photoperiodic flowering, such as PRMTs and SDGs. PRMT6 mediates the interaction between the H3R2me2a system and the NF-CO module to dynamically modulate *FT* expression. Moreover, AtPRMT6 cooperates with its homologous protein AtPRMT4a/4b to repress *FLC*, an inhibitor of *FT*, thus facilitating photoperiodic flowering [137]. Furthermore, AtPRMT6 physically interacts with the positive flowering regulators nuclear factors Y (NF-YC3/9 and NF-YB3) to regulate photoperiodic flowering [137] (Table 1). Collectively, AtPRMT6, NF-YCs, and PRMT4a/4b synergistically regulate photoperiodic flowering via the *CO*-*FT* module or the *FLC*-dependent pathway. SDGs, as the encoders of histone lysine HMTases, may also act in the photoperiodic flowering of multiple species, especially rice and *Arabidopsis*. In short-day rice (*Oryza sativa*), SDG723 (OsTrx1) and SDG724/725 function in photoperiodic flowering as H3K4 and H3K27 HMTase, respectively [138–141] (Table 1). SDG718 (OsiEZ1) and SDG711 (OsCLF), as H3K27 HMTases, function in photoperiodic flowering by repressing *OsLF* (suppressor of *Hd1*) and silencing polycomb [142] (Table 1). Moreover, SDG708/724/725, encoding H3K36-specific HMTase, accelerates rice flowering by stimulating the expression of the *Ehd1*, *Hd3a*, and *RFT1* genes under short/long days [143] (Table 1). Furthermore, SDG712, encoding H3K9-specific HMTase, negatively regulates *Hd1* expression. Subsequently, SDG712 inhibits the expression of florigen genes *Hd3a* and *RFT1* by mediating the H3K9me2 of the two florigen genes, which eventually causes late flowering in rice under short/long days [143]. The *sdg712* mutant flowers early while the *SDG712-OX* line flowers late, which further confirmed the function of SDG712 in photoperiodic flowering [143]. In *Arabidopsis*, AtSDG25 and AtSDG26 encode H3K36 and H3K4/36-specific HMTase, respectively. They participate simultaneously in photoperiodic and vernalization-induced flowering [144, 145] (Table 1). AtSDG25 postpones flowering by activating *FLC* expression, while AtSDG26 advances flowering by binding to the *SOC1* locus [144, 145]. Excitingly, a new study from Guo *et al*., through the isolation and identification of *Arabidopsis* H3K4/K36me2/3 reader MRG1/2, revealed a new mechanism for histone methylation to precisely regulate photoperiodic flowering [146]. They reported that MRG1/2 and histone deacetylase HD2C jointly repress *FT* to set an appropriate flowering time so as to regulate photoperiodic flowering. MRG and *CO* form a protein complex and collaboratively promote the expression of *FT*. Additionally, MRG1/2 can recruit HD2C to the *FT* gene under long days and catalyze the deacetylation of H3K9/23/27ac in the *FT* promoter to inhibit *FT* and thereby delay flowering [146]. These results suggest that histone methylation probably regulates photoperiodic flowering by altering expression of flowering genes such as *FLC*, *CO*, and *FT* or polycomb silencing.

As for RNA, especially non-coding sRNA affects flowering time by directing the methylation status of DNA and histone [147]. The circadian clock, a central player in flowering/meristem transition in bulbous plants, serves as an integrator of low-temperature signals and vernalization-/photoperiod-/meristem transition-related gene expression. In the study of Ben Michael *et al*., long dark cold exposure (photoperiod and vernalization) in *Allium sativum* bulbs induced flowering and bulbing [148]. They shed light on the result that RNA methylation functions in metabolic processes to play general developmental roles in meristem identity affected by vernalization. They identified a series of DEGs related to hormone transport, regulation of gene expression, DNA modification and methylation, cytokinin response, auxin influx, defense response, photoperiod, photosynthesis, and flavonoid metabolism. Overall, RNA methylation or other potential methylation is probably involved in *A. sativum* flowering under co-treatment of vernalization and photoperiod. RNA methylation may be involved simultaneously in both photoperiod- and vernalization-induced flowering. In addition, circadian regulations are required to enable organisms to synchronize physiology with photoperiod. m^6^A is established by ‘writer’ and ‘eraser’ proteins, and its RNA methylation extensively and actively contributes to circadian regulation in seagrasses (*Cymodocea nodosa* and *Zostera marina*) [149]. When the circadian rhythm mechanism senses suitable flowering conditions, the plant will blossom and bear fruit. Seagrasses are a unique group of flowering plants, and whether RNA methylation accelerates flowering by regulating the circadian rhythm in seagrasses remains to be studied’. More regulatory details on whether and how RNA self-methylation directly/indirectly affects photoperiodic flowering in seagrasses are still required. Notably, in *Arabidopsis*, several putative m^6^A erasers belong to the ALKBH family, such as ALKBH9B and ALKBH10B, both of which are functionally characterized in flowering due to their m^6^A demethylase activity [150] (Fig. 4). Fascinatingly, FIONA1-mediated m^6^A methylation may be involved in photoperiodic flowering. FIONA1, a U6 m^6^A methyltransferase from *Arabidopsis*, has been identified as a genetic regulator of the circadian clock. FIONA1 affects day-length-dependent flowering so that *fio1-1* flowers early in a photoperiod-dependent manner [151]. A previous study also revealed that *Arabidopsis fio1-1* mutants show an early-flowering phenotype under long days/short days [152]. However, Wang *et al*. reported that the m^6^A methylation activity of FIONA1 is necessary for phytochrome signaling-dependent photomorphogenesis and photoperiod-independent flowering [151] (Table 1). It is noteworthy that after absorbing different wavelengths of light, photoreceptors can induce plant morphogenesis and have a significant effect on some physiological processes. Phytochrome is a photoreceptor that mediates the reaction of red light and far-red light. FIONA1 is a positive regulator of photomorphology, especially in phytochrome A (Phy A) and Phy B signal transduction in *Arabidopsis* [151]. Therefore, FIONA1-mediated m^6^A methylation might have an effect on floral transition. This remains to be explored. Intriguingly, FIONA1-mediated *FLC* 3′UTR methylation, which regulates *Arabidopsis* flowering, is partially independent of the photoperiodic pathway [153] (Table 1). FIONA1, as an m^6^A writer and floral repressor, stabilizes *FLC* expression by methylating the 3′ end of *FLC* transcripts [153]. The expression of *FT* and *CO* is elevated in *fio1-1* and *fio1-5*, while *fio1 ft* and *fio1 co* flower early under long days/short days [153]. Surprisingly, Xu *et al*. demonstrated that the *Arabidopsis* flowering regulated by FIONA1-mediated m^6^A methylation is closely related to *SOC1*, a key flowering integrator [154] (Table 1). They indicated that FIONA1-mediated m^6^A methylation directly reduces the transcript abundance of *SOC1* and indirectly inhibits the expression of its upstream regulators *SHORT VEGETATIVE PHASE* (*SVP*, a direct repressor of *SOC1*), *CO*, and *FT* to further suppress *SOC1* expression, thus repressing photoperiodic flowering. Mutant *fio1* exhibited global m^6^A mRNA demethylation and an early-flowering phenotype [154]. In addition, the circadian-regulated genes *CCA1* and *LATE ELONGATED HYPOCOTYL* (*LHY*) in *fio1-2* were identified as m^6^A-hypomethylated genes [154]. These two clock genes, whose mRNAs are methylated directly by FIONA1, elevate the expression of *CO* and *FT*, and thereby indirectly participate in *SOC1* suppression [154]. Besides FIONA1, the MT-A70 family methyltransferase METTL4 may also be involved in photoperiodic flowering. Intriguing discoveries by Luo *et al*. revealed that METTL4 acts as the U2 snRNA MTase for *N^6^*-2′-*O*-dimethyladenosine (m^6^Am) *in vivo* and specifically catalyzes the *N^6^*-methylation of Am in ssRNA in *vitro* [155]. The deletion/mutation of METTL4 can promote *Arabidopsis* flowering under long days [155] (Table 1). Notably, in their GO analysis data, a group of DEGs related to photosynthesis and response to low temperature is associated with the regulation of flowering time [155], suggesting that METTL4 as an MTase might involve both photoperiod and vernalization pathways. In general, therefore, it seems that DNA methylation and histone methylation are two main potential methylation types involved in photoperiodic flowering. More detailed information about the involvement of RNA methylation in photoperiodic flowering is needed.

## Conclusions and future outlook

Histone, DNA, and RNA methylation may regulate vernalization and photoperiod-induced flowering through crosstalk with flowering-related regulatory factors (*FLC*, *FT*, *CO* etc.), sRNAs [lncRNA (*COOLAIR*, LDMAR, and osasmr5864m)] and phytohormones. Methylation probably regulates vernalization- and photoperiod-induced flowering simultaneously, and subsequently accelerates plant flowering in coordination. Histone methylation acts on the vernalization and photoperiod pathways via the histone methyltransferase or demethylase and lncRNAs in the lysine and arginine pathways. DNA methylation regulates flowering through the RdDM pathway and plant hormone pathway, and the methylation state carrying vernalization/photoperiod memory may be inherited by subsequent generations, especially plant endogenous methylase mutations (such as *METI*). In addition, based on the evidence that DNA methylation can replace prolonged cold (vernalization) and the photoinduced effect, DNA methylation may replace vernalization and photoperiod as flowering regulators in the future. RNA methylation may regulate vernalization-induced bolting and flowering via RNA methylcytosine transferase. It also participates in photoperiodic flowering by regulating FIONA1 and METTL4 with *N^6^*-methylation activity. However, the exact mechanism by which RNA methylation regulates vernalization- and photoperiod-regulated flowering is still an open question and needs to be further studied. A large number of studies have shown that vernalization- and photoperiod-induced flowering are often accompanied by significant methylation changes. More studies have confirmed that methylation is likely to replace vernalization and photoinduction to promote flowering. Therefore, we propose the feasibility of methylation as a new flowering regulator in the future. Notably, most model plants comply with the above methylation mechanisms of flowering regulation, but there are unequal differences among different species. Methylation reagents may become new flowering accelerators or inhibitors to selectively replace flowering conditions such as low temperature and photoinduction. Methylation-regulated flowering might develop into a novel flowering pathway dependent/independent of other flowering pathways in the future. This methylation regulatory mechanism may lead to the cultivation of plants more adaptable to changing environment.

Flowering is a crucial step in developmental transitions. Methylation in plants may be changed by factors/conditions *in vitro* or *in vivo*, such as methyltransferase/demethylase (Table 1), prolonged cold (vernalization), light quality or intensity, and long/short days. 5-azaC may replace prolonged cold (vernalization) to induce flowering, and the substitution of 5-azaC for the photoinduced flowering effect is not specific to *P. frutescens* [116]. However, 5-azaC cannot replace the photoinduced effect, but only slightly increase the flowering percentage in induced *Chenopodium rubrum* [156]. This means that the substitution of 5-azaC is not suitable for each plant. But it would be viable that the methylation level is changed to regulate flowering time more conveniently and accurately by using these factors/conditions. It will be intriguing to further study the substitutability of flowering conditions by methylation. This may represent the first step in the evolution of methylation treatments as a new practical means of regulating flowering. In human and animal research, huge efforts have been made to develop methylation reagents as clinical drug candidates, such as the development of the DNA methyltransferase inhibitor PRMT5 (one of the most promising anticancer targets). Recent discoveries show that the druggability of histone lysine methylation or its reader is feasible, which means the development of MBD antagonists is feasible [157, 158]. The DNA methyltransferase inhibitor 5-azaC immunoregulates common epithelial cancers [159]. Attenuated fungus treated with 5-azaC increases camptothecine production in host plants [160]. A recent study has proposed the use of demethylation reagents to avoid the decline in the production of secondary metabolites during the maintenance of plant cell cultures *in vitro* [161]. The function of the 5-azaC reagent as a flowering accelerator has been gradually recognized. Methylation/demethylation reagents may show promising strategies for more convenient and finer flowering regulation at molecular level in different species.

However, abnormal changes in DNA methylation may cause developmental abnormalities in plants. For example, a study on the somaclonal mantled African oil palm *Elaeis guineensis* showed that the hypomethylation of Karma LINE retrotransposons of the *EgDEF1* gene results in abnormal fruit with low oil yield [162]. In seed development, the enzymes/proteins involved in active DNA demethylation, including Repressor of silencing 1 (ROS1), Demeter (DME), and downstream proteins of DME, including ZDP, APE1L, and DNA ligase I, were reported to be essential for normal seed development [163]. The mutation of maternal DME leads to failure of maternally expressed genes (MEGs, maternally imprinted genes) activation, and early seed abortion with cessation of embryo growth [164]. In their progeny, homozygous *DME*-null mutants cannot survive, while 50% of the seeds of heterozygous *DME*/*dme-1* and *DME*/*dme-2* plants are aborted. Kim *et al*. indicated that a *Arabidopsis dme-2*-homozygous mutant with a seed abortion rate of 97.1% still produces some seeds and shows early flowering [165]. Single mutants of *APE1L* and *ZDP* show no abnormality, but their double mutants display an embryonic lethal phenotype and produce ~50% abortive seeds after pollination [166]. The expression of imprinted genes in the endosperm of these abortive seeds is downregulated and DNA hypermethylation occurs, such as DNA hypermethylation at selected *MEG* (*FWA* and *FIS2*) promoters. The cytosine methylation in antisense *METI Arabidopsis* decreases, causing many abnormal developmental phenotypes, including reduced apical dominance, smaller plant size, reduced fertility, and changes in leaf size and shape and flowering time [90]. Separating/removing the antisense structure in sexual hybridization cannot completely restore the methylation pattern of the progenies. Similarly, Lang *et al*. found that *SlDML2* mutation leads to genome-wide DNA hypermethylation and fruit ripening inhibition [167]. Moreover, the number of seeds produced in the fruit of the *Sldml2* mutant decreases, indicating that the abnormal increase in DNA methylation may cause the decline in seed yield [167]. Furthermore, exogenous low (30 μM) and high (≥100 μM) concentrations of 5-azaC show extremely different effects on the growth and development of spinach seeds, including seed germination rate, root length, plant height, and flowering time [118]. This shows that 5-azaC-mediated demethylation must be within a certain limit to play a positive regulatory role in plants. Therefore, it is worth considering that the flowering promotion effect mediated by DNA (de)methylation may make it a flowering regulator to some extent, but there is still a big gap in research on whether the progeny will produce abnormal phenotypes, such as abnormalities in flowering, fruit ripening, and seed development. Interestingly, in breeding, the paternal epigenome is the key determinant of the triploid blocking response (failure of endosperm development, arrest in embryogenesis, seed collapse). It was recently discovered that 5-azaC can help *Arabidopsis* bypass triploid seed collapse, and shows stable low-methylation intergenerational inheritance in strong suppressor line (Aza1, 14, 18 and 25; 'suppressor' means the suppression of 5-azaC for triploid blocking reaction) upon CG context, as well as the normal expression of paternally expressed genes (PEGs, the genetic component of triploid seed death) in triploid seeds [168]. Thus, there is some controversy about the effect of applying methylation reagents on growth and development in the progeny. It will be a critical point to explore when and where to use DNA methylation reagents in the future. Of course, even if the use of a methylation reagent results in abnormal seed, flowering and, fruit development in the progenies in some species, it is still available in some suitable scenarios. For example, it is still applicable to some ornamental plants, such as foliage plants, stem plants, and flower plants. Therefore, in the future it is essential to clearly understand and accurately define the use of DNA methylation in the right place at the right time and at the right concentration.

In addition, the following aspects may be worth exploration. The flowering promotion of the autonomous pathway in *Arabidopsis* is unlikely to be due to DNA methylation [169]. Nevertheless, Bäurle and Dean have shown in *Arabidopsis* that FPA (protein containing RRM-type RNA-binding domains) may promote flowering by inhibiting *FLC* at least partly through histone demethylase FLD, and that autonomous pathway-mediated silencing may act through DNA methylation-dependent/independent effects [170]. The age pathway regulated by the miR156-SPL (*SQUAMOSA* promoter binding protein-like) module ensures that plants bloom under non-induced conditions (non-vernalization, non-induced photoperiod) [171, 172]—is methylation involved? Is methylation involved in the GA pathway, the endogenous flowering pathway? What is the relationship among RNA methylation, sRNA, and RdDM in flowering regulation? More molecular evidence of RNA methylation regulating flowering is also one of the next challenges. These derived problems may be worthy of further studies on methylation-regulated flowering.
